# Complaints of Poor Sleep and Risk of Traffic Accidents: A Population-Based Case-Control Study

**DOI:** 10.1371/journal.pone.0114102

**Published:** 2014-12-10

**Authors:** Pierre Philip, Cyril Chaufton, Ludivine Orriols, Emmanuel Lagarde, Emmanuelle Amoros, Bernard Laumon, Torbjorn Akerstedt, Jacques Taillard, Patricia Sagaspe

**Affiliations:** 1 Université de Bordeaux, Sommeil, Attention et Neuropsychiatrie, USR SANPSY 3413, F-33000 Bordeaux, France; 2 CNRS, SANPSY, USR 3413, F-33000 Bordeaux, France; 3 CHU Pellegrin, F-33076 Bordeaux, France; 4 INSERM U897, ISPED, Equipe PPCT, Université de Bordeaux, F-33000 Bordeaux, France; 5 IFSTTAR, TS2, UMRESTTE, F-69500 Bron, France et Université Lyon 1, F-69008 Lyon, France; 6 Stress Research Institute, Stockholm University, Stockholm, Sweden; Osaka University, Japan

## Abstract

**Introduction:**

This study aimed to determine the sleepiness-related factors associated with road traffic accidents.

**Methods:**

A population based case-control study was conducted in 2 French agglomerations. 272 road accident cases hospitalized in emergency units and 272 control drivers matched by time of day and randomly stopped by police forces were included in the study. Odds ratios were calculated for the risk of road traffic accidents.

**Results:**

As expected, the main predictive factor for road traffic accidents was having a sleep episode at the wheel just before the accident (OR 9.97, CI 95%: 1.57–63.50, p<0.05). The increased risk of traffic accidents was 3.35 times higher in subjects who reported very poor quality sleep during the last 3 months (CI 95%: 1.30–8.63, p<0.05), 1.69 times higher in subjects reporting sleeping 6 hours or fewer per night during the last 3 months (CI 95%: 1.00–2.85, p<0.05), 2.02 times higher in subjects reporting symptoms of anxiety or nervousness in the previous day (CI 95%: 1.03–3.97, p<0.05), and 3.29 times higher in subjects reporting taking more than 2 medications in the last 24 h (CI 95%: 1.14–9.44, p<0.05). Chronic daytime sleepiness measured by the Epworth Sleepiness Scale, expressed heavy snoring and nocturnal leg movements did not explain traffic accidents.

**Conclusion:**

Physicians should be attentive to complaints of poor sleep quality and quantity, symptoms of anxiety-nervousness and/or drug consumption in regular car drivers.

## Introduction

Driver sleepiness and fatigue are now considered to be important factors contributing to road traffic accidents [1], [2], [3], [4], [5]. Estimates of the proportion of car crashes attributable to driver sleepiness vary between 6 and 20% in Western countries according to the type of roads and countries.

Ten years ago in New Zealand, Connor et al. [6] assessed the relationship between driver sleepiness and the risk of car accidents in a population-based case-control study that compared 571 car drivers involved in crashes (in which at least one occupant was admitted to hospital or killed) with 588 representative drivers recruited while driving on public roads. The authors found that an increased risk of an injury crash was associated with acute subjective sleepiness immediately before the crash or survey (sleepiness at the wheel), and two determinants of it: acute sleep deprivation (i.e. driving after five hours of sleep or fewer) and time of the day (i.e. driving between 2 am and 5 am). In contrast, no increase in risk was associated with measures of excessive daytime sleepiness, chronic partial sleep deprivation or heavy snoring. Interestingly, sleep-related questions concerned only a small part of the interview and the study did not focus on complaints related to sleep quality and quantity.

More recently, two French epidemiological studies confirmed that sleepiness at the wheel was associated with a higher risk of road accidents [4], [5]. They also identified insomnia and mental disorders as new factors associated with an increased risk of road accidents.

More than ten years have passed since the Connor study [6] which has not yet been replicated. In addition, no study to our knowledge has explored the involvement of additional factors such as mental disorders or insomnia in the increase in crash risk by using the same method. Therefore we designed a case-control study whose objective was to examine the relationship between the risk of severe road accidents and classical factors (i.e., sleepiness at the wheel, sleep duration) or new factors unexplored by Connor's study such as sleep complaints and mental disorders.

## Methods

We conducted a case-control study between June 15^th^, 2011 and October 31^st^ 2012. Data were collected in a large agglomeration (Bordeaux, 750,000 inhabitants) and in a small-sized city (Libourne, 30,000 inhabitants) in the Gironde department, Aquitaine, southwest France. The regions include intra-city road and rural areas.

The study population included 544 participants who drove a light 4-wheel vehicle (272 cases and 272 controls). Drivers of trucks, taxis and emergency vehicles were excluded from the study because they may be considered as professional drivers.

The study was approved by the local ethics committee (consultative committee for the protection of persons participating in biomedical research [CPP Bordeaux]). Informed consent by participants was not required by the ethics committee because this study was considered as an epidemiological study and not as an interventional one. The database was anonymous according to the recommendations of the French Data Protection Authority which insures the ethical usage of data collected for scientific purpose.

### Selection of cases

We included drivers who were admitted to emergency units after a road traffic accident. They were informed about the aim of the study and interviewed about the circumstances surrounding the accident by a clinical research assistant face-to-face (n = 227/272, 83.5%) or by telephone at home (n = 45/272, 16.5%) when hospitalization was too short.

Interviewers had received intensive training with a physician to be able to conduct a clinical interview focused on sleep complaints and sleep disorders. In total, 272 cases (Bordeaux, n = 224, 82.4% and Libourne, n = 48, 17.6%) were included in the study.

### Selection of controls

Controls were drivers interviewed during road checks randomly carried out by the police forces. The selection of controls was matched on time of day of the accidents with controls. The police stopped cars one by one in intra-city road and rural areas and conducted their routine inspection, which included inspecting the driver's license and sometimes requesting an alcohol breath test. Afterwards a clinical research assistant asked the drivers if they agreed to participate in the present study. If so, they were interviewed at the roadside. In total, 272 controls were included in the study.

### Questionnaire administration

Participants (i.e., cases and controls) were asked by a research assistant trained in sleep medicine and clinical evaluation to answer a 20-minute questionnaire based on Connor's methodology [6] at the hospital within the 24 hours following the accident or when stopped by the police. The questionnaire included questions about demographics, working hours, driving habits, accident circumstances, acute measures of sleepiness (including sleep in the last 24 hours), sleep hygiene (sleep-wake schedules), chronic measures of sleepiness (Epworth Sleepiness score [7]) and sleep disorders (e.g., symptoms of insomnia, sleep apnea and restless legs) in the last three months, self-rated health status and alcohol/drug intake in the last 24 hours. The questions related to how the cases felt immediately before the accidents were worded for the controls as “immediately before being stopped by the police”.

### Calculation of sample size

Considering the association between the risk of car crash injury and a Stanford Sleepiness score of more than 3 (4–7), the sample size to achieve a sufficient statistical power to demonstrate an OR of 8.2 and more, as estimated by Connor and colleagues [6] (with an exposure rate among controls of 1%), had to include 90 subjects in each case and control group, with a first and second order risk of 5% (alpha) and 20% (beta). Our sample size therefore allowed for further adjustment on potential confounders.

### Statistical analyses

Quantitative variables were expressed as mean and standard deviation (SD), and qualitative variables were expressed as relative frequency. The Khi^2^ or t-test were used to show significant differences between cases and controls (p<.05).

All variables associated with being a case (i.e., having a driving accident) were initially examined separately using univariate models.

Multivariate logistic regression analyses were performed for all variables that showed a significant association in univariate models (p<0.05) to control for confounding factors. The referent group for each factor was selected as drivers thought to be at the lowest risk of accidents.

A significant association was found between being a case (i.e., having a driving accident) and the following variables:

Demographic variables: Age, gender and marital status;

Driving variables: Time since acquisition of driver's license, kilometers driven per year and type of road;

Legal drug consumption: Medication in the last 24 h, taking hypnotics on the previous day, taking antidepressants on the previous day, number of medications taken with pictogram (medications were labeled with risk levels in the French classification system from 0 “no risk” to 3 “high risk”) in the last 24 h, medications with pictograms 2 (be very careful) or 3 (danger: do not drive) in the last 24 h, taking a treatment against insomnia in the previous week, taking a treatment against anxiety in the previous week, taking a treatment against depression in the previous week and treatment for a sleep disorder;

Behavioral variables: Taking a break during the journey, adjusting the radio, GPS or dialing a phone number, and looking at something in the environment;

Sleep-related and psychiatric variables: Epworth Sleepiness Scale (ESS) scores [7], total sleep time in the last 24 h, sufficient sleep in the previous night, having symptoms of anxiety or nervousness on the previous day, symptoms of depression on the previous day, experience of occupational stress on the previous day, experience of personal stress on the previous day, sleepiness at the wheel 10 min just before the accident, having a sleep episode just before the accident, difficulty falling asleep, repeated awakenings, premature awakening, not being refreshed by sleep, leg movements during sleep in the last three months, sleeping 6 hours of nocturnal sleep or fewer in the last three months, needing to fight against sleep to keep awake, quality of sleep and quantity of sleep.

Age, marital status, holding a driving license, kilometers driven per year, type of road, and number of medications in the last 24 h were categorized for statistical analysis. Taking a break during the journey was dichotomized as yes or no. ESS scores were classified as 0–10 or >11. Total sleep time in last 24 h was dichotomized as fewer than 6 hours or more than 6 hours. Sufficient sleep in the previous night, having symptoms of anxiety or nervousness on the previous day, experiencing symptoms of depression on the previous day, experience of occupational stress on the previous day, experience of personal stress on the previous day, taking hypnotics on the previous day, and taking antidepressants on the previous day were dichotomized as yes or no. Number of medications taken with pictogram in the last 24 h was categorized as 0, 1, 2 and ≧3. Medications with pictograms 2 or 3 in the last 24 h, taking a treatment against insomnia in the previous week, taking a treatment against anxiety in the previous week and taking a treatment against depression in the previous week were dichotomized as yes or no. Sleepiness at the wheel 10 min just before the accident was categorized as 1–4, 5–6 and 7–9. Adjusting the radio, GPS or dialing a phone number, looking at something in the environment and having a sleep episode just before the accident were dichotomized as yes or no. Difficulty falling asleep, repeated awakenings, premature awakening, not being refreshed by sleep, leg movements during sleep, sleeping 6 hours or fewer and needing to fight against sleep to keep awake in the last three months were dichotomized as never, rarely/sometimes, often/very often. Quality of sleep in the last three months was dichotomized as very/pretty good, neither good nor bad, pretty/very bad. Quantity of sleep in the last three months was dichotomized as definitely/broadly enough, rather little, clearly/definitely not enough. Treatment for a sleep disorder was dichotomized as yes or no.

For statistical analyses, we used self-reported data on alcohol consumption rather than blood alcohol concentrations measured at the emergency unit because the former are far more complete.

Statistical tests of odds ratios (ORs) were based on Wald statistics. Odds ratios and their 95% confidence intervals (CIs) are presented to show the association.

All analyses were performed with the SPSS statistical software package (SPSS, version12.0, Chicago, IL, USA).

## Results

### Population

We assessed 840 cases for eligibility. Out of these 840, 53 were unable to answer (severe injury), 18 refused to participate and 497 subjects were not interviewed (hospitalization <2 h). The final sample for analysis comprised 272 patients (32% of the 840 eligible drivers). Of 272 cases, more than half were females (57.3%). The mean age was 39.8±16.9 for cases and 43.9±16.0 for controls. The Body Mass Index (BMI) ranged from 15.6 to 44.4 kg m^−2^ for cases and from 16.8 to 47.8 kg m^−2^ for controls (Table 1).

**Table 1:** Demographic characteristics of cases (i.e., drivers admitted to emergency unit at hospital) and controls (i.e., drivers interviewed during road checks carried out by the police).

Characteristics	Percent or Mean ± SD		Khi^2^ or T-test
	Cases (n = 272)	Controls (n = 272)	*p* value
Age (years) (%)			.01
18–30	38.6	24.6	
31–50	38.6	42.5	
51–65	12.9	20.6	
>65	9.9	12.1	
Female (%)	57.3	42.6	.001
BMI (kg m^−2^)	23.8±4.0	24.5±4.1	ns
Marital Status (%)			.01
Married	50.7	67.1	
Single	37.9	26.8	
Separated, divorced or widowed	11.4	7.7	
Occupational status (%)			.001
Active	67.0	72.5	
Retired	11.7	17.2	
Student	5.9	1.8	
Non-active	9.2	3.7	
Disabled person	4.4	1.1	
Other	1.5	3.3	
Working period (%)			ns
Diurnal work	57.9	63.4	
Shift work without night shift	5.5	3.7	
Shift work with night shift	6.2	5.1	
Nighttime work	0.7	1.1	
Working hours per week (h)	39.2±11.9	38.9±9.9	ns
Having a driving license (years)			.05
0–2	32.3	22.1	
>2	65.1	75.7	
Kilometers driven per year (%)			.001
0–5 000	25.0	12.1	
>5 000	56.6	77.9	

BMI, body mass index.

Column totals may differ owing to missing data.

### Univariate analyses

#### Sleep complaints

Univariate analysis showed that case drivers reported more nocturnal sleep complaints and EDS than control drivers (Table 2). 26.5% of cases and 20.6% of control drivers reported sleeping fewer than 6 hours per night in the last three months.

**Table 2:** Sleepiness assessment, symptoms of sleep disorders, psychiatric symptoms, medication consumption and driving conditions for cases (i.e., drivers admitted to emergency unit at hospital) and controls (i.e., drivers interviewed during road checks carried out by the police).

Characteristics	Percent or Mean ± SD		Khi^2^ or T-test
	Cases (n = 272)	Controls (n = 272)	*p* value
Degree of sleepiness at the wheel 10 min before accident or interview			.01
1–4	89.0	95.6	
5–6	4.4	3.7	
7–9	5.1	0.7	
Having a sleep episode just before accident or interview	6.2	1.1	.001
Being involved in an accident due to sleepiness in the previous year	3.3	2.3	ns
ESS			.05
0–10	86.4	92.3	
>11	13.6	7.7	
Total sleep time in last 24 h less than 6 hours	22.4	15.4	.05
Sufficient sleep in previous night	74.6	82.0	ns
Had a nap during the day	7.7	10.3	ns
Driving duration before accident or interview (min)	22±24	28±40	.056
Quality of sleep			.001
Very/pretty good	73.5	83.8	
Neither good nor bad	10.3	8.2	
Pretty/very bad	10.7	6.6	
Quantity of sleep			.001
Definitely/broadly enough	59.2	76.8	
Rather little	8.1	10.3	
Clearly/definitely not enough	32.4	13.0	
In the last three months, experienced any of the following symptoms (often/very often)			
Difficulty falling asleep	29.8	11.8	.001
Disturbed/restless sleep	26.1	15.8	.01
Repeated awakenings	25.0	12.5	.001
Premature awakening	26.1	16.5	.05
Not being refreshed by sleep	21.7	8.1	.001
Heavy snoring	13.6	16.2	ns
Respiratory interruptions during sleep	1.1	1.5	ns
Leg movements during sleep	10.7	5.5	.05
Slept 6 hours or fewer	26.5	20.6	.001
Sleepiness at work/leisure	8.1	5.1	.01
Involuntary sleep episodes at work/leisure	3.3	0.7	.05
Need to fight against sleep to keep awake	6.6	2.9	.001
On the previous day, symptoms of			
Persistent fatigue	16.5	12.9	ns
Severe pain	3.7	6.2	ns
Fever/flu symptoms	1.8	1.5	ns
Mental fatigue	18.7	10.7	.01
Anxiety/nervousness	27.6	14.7	.001
Depression/low mood	9.9	4.0	.05
On the previous day, experience of			
Occupational stress	12.9	21.0	.01
Personal stress	19.8	8.8	.01
On the previous day, taking			
Analgesic/antipyretic medication	10.6	10.6	ns
Sedatives	8.8	0.4	.001
Hypnotics	4.0	0.4	.05
Antidepressant medication	9.6	0.4	.001
Number of medications in the last 24 h			.001
0	34.9	51.1	
1	28.7	32.0	
2	16.9	11.8	
≥3	19.5	5.1	
Medications with pictograms 2 or 3in the last 24 h	19.1	5.9	.001
In the previous week, treatment against			
Insomnia	8.8	0.4	.001
Sleep apnea	1.1	0.0	ns
Restless legs syndrome	0.4	0.0	ns
Anxiety	11.8	1.8	.001
Depression	10.7	0.4	.001
Neurological disease	1.1	0.4	ns
Psychiatric disease	1.8	0.4	ns
Chronic pain	6.2	2.2	.05
Headache	9.2	7.7	ns
Pericardial disorder	7.0	2.2	.05
Hypertension	9.6	8.5	ns
Treatment for a sleep disorder	19.8	8.8	.001
Taking a break during the journey	9.9	27.9	.001
Alcohol/illicit substance intake	23.9	35.3	.01
Type of road			.05
Intra-city road	46.7	57.7	
2 lanes country roads	41.2	36.0	
Highway	11.8	6.2	
Time of day of accident or interview			ns
Day	70.6	70.6	
Evening	11.8	11.8	
Night	17.6	17.6	
Main reasons for driving on road			.001
Occupational	5.1	16.5	
Home – workplace	26.7	17.9	
Other	68.1	65.2	
Undertaking distracting activities			
Adjusting the radio, GPS or dialing phone number	1.8	6.6	.01
Reaching for object in the car	0.7	0.4	ns
Talking on mobile phone	0.0	2.6	.01
Reading a map (or something else)	1.5	0.0	ns
Looking at something in the environment	4.4	0.7	.01
Disturbed by a passenger	1.1	0.7	ns

50.9% of these drivers reporting 6 hours of sleep per night or fewer during the last 3 months also reported insomniac complaints (experienced often/very often in the last three months: difficulty falling asleep, or disturbed/restless sleep, or repeated awakenings or premature awakening), while 49.1% of the drivers reported a short duration of sleep with no nocturnal complaints, referring more to an insufficient sleep syndrome.

#### Driving accidents and sleepiness

Most cases had minor injuries while severe injuries that prevented interviewing were rare (1.9%). Daily monitoring allowed the inclusion of 71.2% of the potential cases who were admitted to the emergency unit. The most common accidents were single-vehicle accidents (38.2%), collision with vehicle in the rear (23.2%), collision at intersection (12.1%), and collision with vehicle in the front (8.1%). 46.7% of the accidents occurred on intra-city roads, 41.2% on 2 lanes country roads and 11.8% on highways or four-lane roads. The peak accident time was between 06.00 and 12.00 h (44.1% of accidents). Conversely, nighttime accidents occurring between midnight and 06.00 h were rare (6.6% of accidents).

6.2% of the cases experienced a sleep episode just before the accident versus only 1.1% of the 272 control drivers before the interview. 10.3% of the cases reported that sleepiness was a factor contributing to their accident (Table 2).

### Multivariate regression analysis of factors explaining road traffic accidents

#### Sociodemographic factors and driving behaviors

Compared with our reference group (51–65-year-old drivers), 18- to 30-year-old drivers' accidental risk was associated with a 3.30-fold increase (CI 95%: 1.12–9.73, p<0.05). Driving on highway/four-lane roads compared to other types of road was also associated with accidental risk (OR 3.77, CI 95%: 1.58–9.02, p<0.01). Drivers who did not take a break during the journey were at higher risk of accidents than those who did (OR 4.04, CI 95%: 2.00–8.18, p<0.001). Experiencing a sleep episode at the wheel just before the accident was associated with a 9.97-fold increase (CI 95%: 1.57–63.50, p<0.05) in driving accident probability.

#### Medical treatments

Drivers who took more than 2 medications in the last 24 h were at higher risk of being involved in a road traffic accident (OR 3.29, CI 95%: 1.14–9.44, p<0.05). Note that the most common associations for a major risk factor of accidents were by ascending order: antidepressant medication and sedatives 15.8% of cases, antidepressant medication and analgesic/antipyretic medication 9.2% of cases, antidepressant medication and hypnotics 7.9% of cases, analgesic/antipyretic medication and sedatives 6.6%, sedatives and hypnotics 5.3% of cases, and hypnotics and analgesic/antipyretic medication 5.3% of cases.

#### Sleep and anxiety complaints

Drivers who claimed to have very poor quality of sleep had a 3.35-fold increase in risk (CI 95%: 1.30–8.63, p<0.05). Drivers who reported 6 hours or fewer of nocturnal sleep during the last 3 months were at significantly increased risk compared to those who had more than 6 hours (OR 1.69, CI 95%: 1.00–2.85, p<0.05). Interestingly, anxiety-nervousness on the previous day was also associated with accidental risk (OR 2.02, CI 95%: 1.03–3.97, p<0.05) but occupational stress was a protective factor (OR 3.60 CI 95%: 1.75–7.41, p<0.001) (Table 3).

**Table 3:** Multivariate logistic regression results for prediction of being a case (i.e., having a driving accident).

		Driving accident			
	Total *n*	Yes *n*	%	Odds Ratio (95%CI)	*p* value
Age (years)					
51–65	90	35	38.9	Referent	
18–30	172	105	61.0	**3.30** (1.12–9.73)	.05
31–50	221	105	47.5		ns
>65	60	27	45.0		ns
Type of road					
Intra-city road	284	127	44.7	Referent	
Highway/four-lane roads	49	32	65.3	**3.77** (1.58–9.02)	.01
Ordinary	210	112	53.3		ns
Medication in the last 24 h					
0	319	133	41.7	Referent	
1	120	63	52.5		ns
2	50	35	70.0	**3.29** (1.14–9.44)	.05
≥3	49	39	79.6	**3.46** (1.11–10.75)	.05
Break during the journey					
Yes	103	27	26.2	Referent	
No	439	243	55.3	**4.04** (2.00–8.18)	.001
Symptoms of anxiety or nervousness on the previous day					
No	428	197	46.0	Referent	
Yes	115	75	65.2	**2.02** (1.03–3.97)	.05
Experience of occupational stress on the previous day					
Yes	92	35	38.0	Referent	
No	448	237	52.9	**3.60** (1.75–7.41)	.001
Having a sleep episode just before the accident					
No	511	242	47.3	Referent	
Yes	20	17	85	**9.97** (1.57–63.50)	.05
Sleeping 6 h or fewer in the last three months					
≧6 h	208	77	37.0	Referent	
<6 h	336	195	58.0	**1.69** (1.00–2.85)	.05
Quality of sleep in the last three months					
Very/pretty good	410	181	44.1	Referent	
Neither good nor bad	76	46	60.2	**2.07** (0.99–4.32)	.052
Pretty/very bad	58	45	77.6	**3.35** (1.30–8.63)	.05

Figures are adjusted odds ratios and 95% confidence intervals (CI) for multivariate model *.

* Logistic regression analysis included age group, gender, kilometers driven per year, years of having a driving license, type of road and medication in the last 24 hours.

Heavy snoring, nocturnal leg movements and chronic daytime sleepiness measured by the ESS did not remain in the model.

## Discussion

This controlled study confirms that sleepiness at the wheel is the strongest predictor of road traffic accidents and that usual short sleep duration (<6 hr), poor sleep quality perception and medicinal drug consumption are associated with the occurrence of road accidents. Interestingly, sleep-related accidents occurred preferentially on the highway irrespective of the time at which the trip occurred. This could be explained by a higher legal speed limit on highways even if the French highways are the safest network for driving (vs. intra-city roads and 2 lanes country roads). Not taking a break during the journey also increases the risk of accidents. Type of roads and how the trip is organized are also clearly associated with the occurrence of sleep-related accidents.

Finally, we confirm that accidents mainly involve young people (i.e., 18–30 years old).

Our investigations confirm that falling asleep at the wheel is the strongest sleep-related factor associated with accident risk, as shown in previous studies [4], [5], [6]. Even if this case control study was not designed to analyze the prevalence of sleep-related accidents, our results corroborate recent French studies showing a prevalence of 6 to 10% of accidents due to sleepiness in a representative population of drivers [4] and in registered highway drivers [5].

We also found that less sleep and impaired quality of sleep over the last 3 months were associated with car accidents. There was almost a two-fold increased risk in drivers who reported 6 hours or fewer of nocturnal sleep in the last 3 months. The question arises whether drivers reporting short sleep duration were in fact insomniacs not perceiving their sleep or chronically sleep-deprived subjects suffering from poor sleep hygiene. Interestingly, a little more than half of the drivers reporting insufficient sleep also reported sleep complaints (i.e. Difficulty falling asleep and/or Repeated awakenings and/or Premature awakening). Even if these criteria partially fit the definition of chronic insomnia (based upon DSM-5 or ICSD-3 classifications), our findings suggest that this disorder might be an important factor helping to explain road accidents.

Interestingly, anxiety-nervousness was independently associated with accident risk while occupational stress was not associated with accidents. Emotional states such as anger or anxiety lead to negative and dangerous driving pattern [8]. Alternatively, the anxiety-nervousness itself may be associated with an increased risk of motor vehicle collisions [9]. Moreover, in a previous study, we have shown a significant association between accident risk and intense mind-wandering [10]. Mind-wandering just before the crash could jeopardize the ability of drivers to incorporate information from the environment. In a recent study [11], both feelings of sadness and anxiety were significantly and positively associated with mind-wandering to relevant life concerns, suggesting that aversive feelings tend to precede mind-wandering to current concerns. Therefore, we suggest that anxiety–nervousness might enhance mind-wandering and/or rumination leading to an increased risk. This finding is important because most studies to date have analyzed the use of psychotropic drugs such as benzodiazepine and accident risk but not the reasons why the drug was taken. Anxiety-nervousness and sleep complaints therefore both appear to be risk factors independently of CNS treatment.

In our study, experience of occupational stress on the previous day appeared as a protective factor. A study [12] found a protective effect of moderate level of psychological distress against crash in young drivers. Interestingly, our group has published a study showing that conversation with a passenger may contribute to safer lane-keeping when driving under a benzodiazepine [13]. These results are consistent with the hypothesis that moderate level of distress or occupational stress may have a protective effect in encouraging more cautious driving behaviors and resulting in fewer driving accident.

Finally, our study shows that having taken 2 or more medications is associated with an increased risk of accidents. Patients treated for illness, injury and/or pain have a higher risk of traffic accidents than patients in good health. We do not know whether side effects of drugs are really involved in this increase in the accident rate or whether the disease itself and/or the pain are confounding factors. Our results confirm that taking psychotropic substances, identified as level 2 or 3 medications, is a risk factor of having an accident. These results illustrate the risk related to level 2 and 3 medications, especially for anti-depressant and hypnotic treatments. We confirm that the ESS is an insensitive measure not associated with accident risk, as previous authors have shown [6], [14]. Unlike for chronic daytime sleepiness, our study shows that situational assessments (e.g., did you have a sleep episode just before the accident?) are more appropriate for evaluating accident risk. It is therefore important to consider sleepiness at the wheel and not simply EDS when evaluating the driving risk in sleepy patients.

Our study has some limitations that may have introduced biases. The low response rates (cases 32%, controls 90%) and lack of available information about those who were not interviewed or declined to participate give rise to a sampling bias. Furthermore, the relatively small number of cases compromised the precision of some estimates of interest. However, the vast majority of accidents that were not investigated were due to the fact that the drivers involved were not severely injured and promptly left hospital.

The suspected risk factors associated with accidents were entirely based on self-reporting, thus increasing the risk of a recall bias and misclassification. Some factors such as sleep disorders and medications may have been underestimated. Alcohol/illicit substance intake and the use of devices while driving (radio, GPS, phone) were certainly under-reported among the cases because of their illegal status. Similarly, consumption of medication reported by the controls may have been underestimated given the non-medical interview environment. The responses of control drivers may have been somewhat affected by the presence of police at the roadside interviews. Further studies should try to control additional contributing factors to accidents such as speed of driving, season, visibility and road status.

The present findings should contribute to knowledge about basic road safety by providing a better understanding of the dangers of sleepiness at the wheel and its behavioral, pathological and iatrogenic origins.

Road safety campaigns should encourage drivers to have good sleep hygiene all year long; Physicians should advice their patients reporting poor sleep hygiene on driving risks independently from their medical treatments.
